# Supplementary material for: Novel Driver Strength Index highlights important cancer genes in TCGA PanCanAtlas patients
Source: PeerJ. 2022 Aug 11;10:e13860. doi: 10.7717/peerj.13860 (PMC9375969; doi:10.7717/peerj.13860)
Supplement: Supplemental Information 2 [file peerj-10-13860-s002.zip › COHORTS/genes plots/cohorts/2021_8_16_14_9_distribution_cohorts_males_BLCA.pdf]

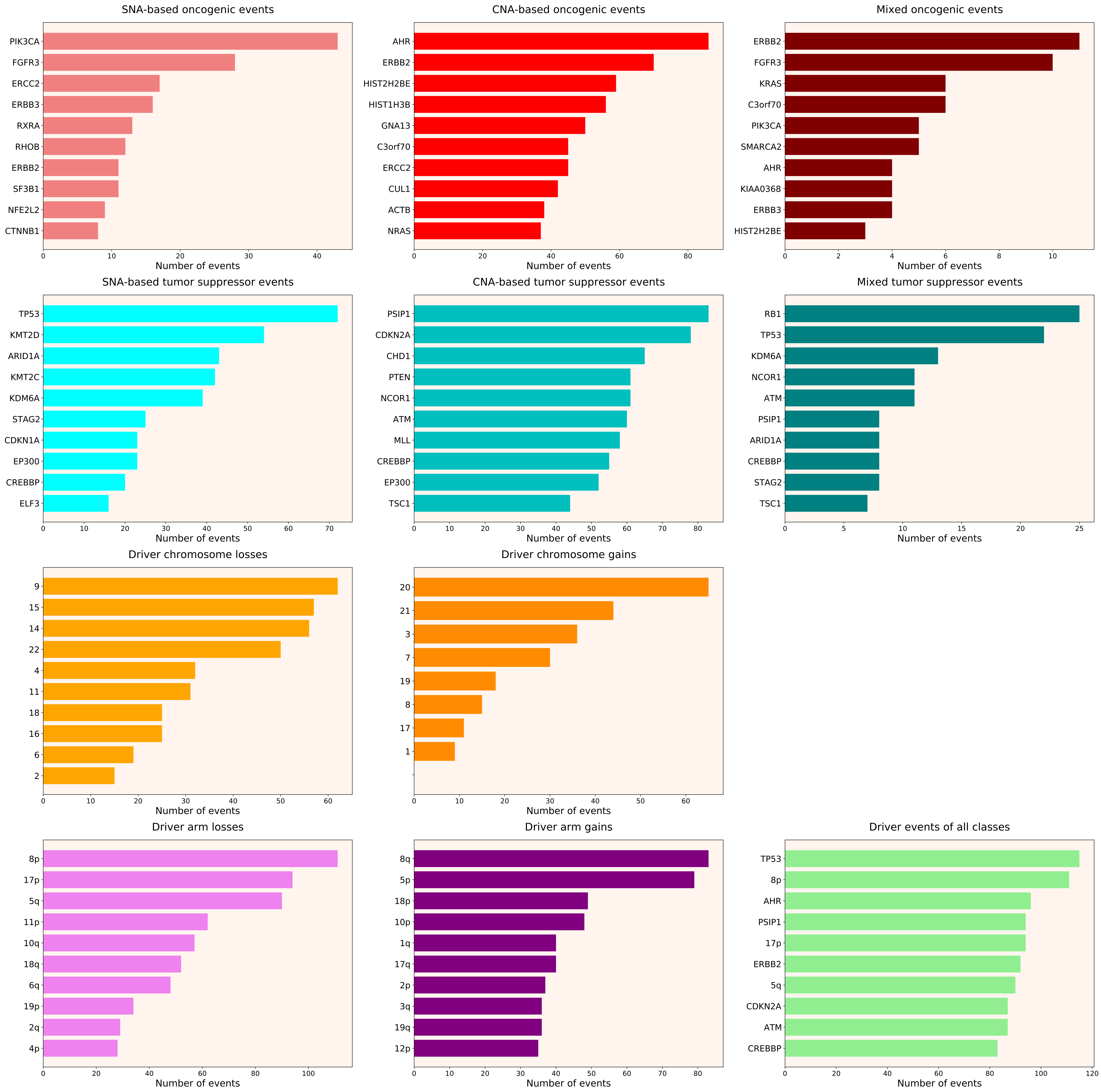

SNA-based oncogenic events
PIK3CA
FGFR3
ERCC2
ERBB3
RXRA
RHOB
ERBB2
SF3B1
NFE2L2
CTNNB1
0 10 20 30 40
Number of events
CNA-based oncogenic events
AHR
ERBB2
HIST2H2BE
HIST1H3B
GNA13
C3orf70
ERCC2
CUL1
ACTB
NRAS
0 20 40 60 80
Number of events
Mixed oncogenic events
ERBB2
FGFR3
KRAS
C3orf70
PIK3CA
SMARCA2
AHR
KIAA0368
ERBB3
HIST2H2BE
0 2 4 6 8 10
Number of events
SNA-based tumor suppressor events
TP53
KMT2D
ARID1A
KMT2C
KDM6A
STAG2
CDKN1A
EP300
CREBBP
ELF3
0 10 20 30 40 50 60 70
Number of events
CNA-based tumor suppressor events
PSIP1
CDKN2A
CHD1
PTEN
NCOR1
ATM
MLL
CREBBP
EP300
TSC1
0 10 20 30 40 50 60 70 80
Number of events
Mixed tumor suppressor events
RB1
TP53
KDM6A
NCOR1
ATM
PSIP1
ARID1A
CREBBP
STAG2
TSC1
0 5 10 15 20 25
Number of events
Driver chromosome losses
9
15
14
22
4
11
18
16
6
2
0 10 20 30 40 50 60
Number of events
Driver chromosome gains
20
21
3
7
19
8
17
1
0 10 20 30 40 50 60
Number of events
Driver arm losses
8p
17p
5q
11p
10q
18q
6q
19p
2q
4p
0 20 40 60 80 100
Number of events
Driver arm gains
8q
5p
18p
10p
1q
17q
2p
3q
19q
12p
0 10 20 30 40 50 60 70 80
Number of events
Driver events of all classes
TP53
8p
AHR
PSIP1
17p
ERBB2
5q
CDKN2A
ATM
CREBBP
0 20 40 60 80 100 120
Number of events
